# Interesting Manner of Extension of Ringworm (Tricophyton Tonsurans)

**Published:** 1880-04

**Authors:** Chas. A. Meyer


### IV.—INTERESTING MANNER OF EXTENSION OF RING-WORM (TRICOPHYTON TONSURANS).

By CHAS. A. MEYER, D. V. S.

In October, 1879, I was asked to look at a horse with skin disease; one which had been lately purchased, but whose skin had broken out in round and scaly patches; the patches were one quarter to one half inch in diameter. My diagnosis was ring-worm, and I placed the animal under treatment; in a few days other cases were shown me, and in a little over a week twenty three horses were affected; they were saddle horses, and the disease baffled me for a while. I used the various mercurial preparations; gave sun baths; but there was very little change for the better. I put the horses under an alkaline treatment, both externally and internally, but of no avail; my patches would continue to form, the hairs break off, and the patches were covered with a thick scurf. I then had the stable disinfected, harness and blankets washed and hung in the sun. I then used a preparation of carbolic acid one part to four parts glycerine, with directions to have the horses washed with soap and warm water, and when dry to touch the parts affected twice per day with the above mixture. On the other cases I used crude petroleum. In a few days I had a change for the better; on those where carbolic acid and glycerine were used the cases recovered in from nine to fourteen days; on those where I used crude petroleum they recovered in from four to seven days. When I saw that the disease was under control I had the saddles re-lined and the trappings touched with a strong solution of carbolic acid; in six weeks the disease was entirely eradicated. I would state that care should be used in employing the petroleum, as when applied to too large a surface it acts like a vesicant. Only the affected spots need to be saturated with the oil. One groom, disregarding his instructions, used it over a large surface, and the hairs all came out, although a new growth of hair took place. My experiments shows that petrolenm is the most efficient parasiticide for Tinea tonsurans, and makes a quicker cure. The parts of the body most affected were the face, neck, shoulders and back. Strange to say, either only the left side of the twenty-three horses was affected, or, where both sides were affected, the left showed the disease most intensely. This phenomenon I attributed to the fact that the horse first affected stood near the entry, and that a current of air, in carrying the germs, deposited them chiefly or exclusively on the most exposed side of the animals.

